# Oil palm monoculture induces drastic erosion of an Amazonian forest mammal fauna

**DOI:** 10.1371/journal.pone.0187650

**Published:** 2017-11-08

**Authors:** Ana Cristina Mendes-Oliveira, Carlos A. Peres, Paula Cristina R. de A. Maués, Geovana Linhares Oliveira, Ivo G. B. Mineiro, Susanne L. Silva de Maria, Renata C. S. Lima

**Affiliations:** 1 Laboratory of Ecology and Zoology of Vertebrate, Institute of Biological Science, Federal University of Pará, Belém, Pará, Brazil; 2 Centre for Ecology, Evolution and Conservation, School of Environmental Sciences, University of East Anglia, Norwich, Norfolk, United Kingdom; Università degli Studi di Napoli Federico II, ITALY

## Abstract

Oil palm monoculture comprises one of the most financially attractive land-use options in tropical forests, but cropland suitability overlaps the distribution of many highly threatened vertebrate species. We investigated how forest mammals respond to a landscape mosaic, including mature oil palm plantations and primary forest patches in Eastern Amazonia. Using both line-transect censuses (LTC) and camera-trapping (CT), we quantified the general patterns of mammal community structure and attempted to identify both species life-history traits and the environmental and spatial covariates that govern species intolerance to oil palm monoculture. Considering mammal species richness, abundance, and species composition, oil palm plantations were consistently depauperate compared to the adjacent primary forest, but responses differed between functional groups. The degree of forest habitat dependency was a leading trait, determining compositional dissimilarities across habitats. Considering both the LTC and CT data, distance from the forest-plantation interface had a significant effect on mammal assemblages within each habitat type. Approximately 87% of all species detected within oil palm were never farther than 1300 m from the forest edge. Our study clearly reinforces the notion that conventional oil palm plantations are extremely hostile to native tropical forest biodiversity, which does not bode well given prospects for oil palm expansion in both aging and new Amazonian deforestation frontiers.

## Introduction

Some 20% of the ~5 million km^2^ Brazilian Amazon has already been deforested since 1970[1]. Anthropogenic land-use, such as livestock ranching, timber extraction, mining and more recently, large-scale intensive agriculture, has historically driven economic development across the region, which is reflected in a regional-scale growth in Gross Domestic Product (GPD) ~1.4% higher than that of the rest of Brazil[2]. However, these development frontiers have brought unprecedented environmental impacts to the region, including elevated deforestation, greenhouse gas emissions, forest degradation, defaunation, soil erosion, and wholesale indiscriminate spread of agricultural pesticides, all of which are unaccounted for in country-scale measures of wealth[2][3].

Oil palm plantations have become one of the most financially attractive crops in Amazonia, not least because of the introduction of government-subsidized biodiesel to the Brazilian energy grid since 2010[4] and restrictions imposed on biofuel cropland expansion in Southeast Asia[5]. Palm oil extracted from *Elaeis guineensis* (Jacq.), yields higher productivity than other sources of biofuel[6][7] but also satiates the burgeoning demand for this versatile product from food, chemical, and cosmetics industries. Brazilian Amazonia currently has one of the world’s largest potential areas for oil palm expansion (~2.3 million km^2^), related to climatic, edaphic and topographic crop suitability[8][9]. The Brazilian government has actively encouraged oil palm expansion, which is extolled as a new opportunity to bring about socioeconomic development and recovery of degraded areas in Amazonia. New state-level legislation has been sanctioned to regulate oil palm plantations on forest areas (including secondary and logged primary forest), or on fallow land (e.g., *Instrução Normativa* SEMAS/PA/2011). In particular, the State of Pará has legally proposed that silviculture of exotic species, such as oil palm, should count towards the restoration of up to 30% of natural forest set-asides within all private landholdings, which is mandatory under Brazilian environmental legislation (Federal Law No. 12.651/2012). Also, low land prices, cheap labor, cheap energy sources from hydropower infrastructure and government-subsidized road-building[8][10], have further fueled the Amazon’s potential to become the world’s largest oil palm producer within a few decades[6].

Given its economy of scale, oil palm cultivation requires large tracts of land, which has resulted in the conversion of over 14 Mha of forest in Southeast Asia[9][11]. In contrast with the original old-growth forests they replace, these plantations present an uniform habitat and tree age structure[12], changes in soil fertility[13] and in the interaction with soil microbes[14], a narrow spectrum of food resources, low-density understory, exposed soils, reduced leaf litter[15][16], highly volatile microclimate[17], and a much lower discontinuous canopy[18]. Faunal diversity responses to these structural changes depending on both the landscape context of plantations and species ecological plasticity in terms of tolerance to a severely modified habitat[19][20][21]. Ecological studies addressing multiple taxa, including birds[20][21][22][23][24], reptiles[20], non-flying small mammals[25], bats, primates[26], butterflies[27], ants[28] and aquatic invertebrates[29], have all shown that oil palm plantations are significantly more depauperate than adjacent primary forests, even if these had been selectively-logged[12].

The total amount and distribution of remaining natural forest cover are critical determinants of the fraction of native biodiversity, retained within agricultural landscapes[30][31]. However, physical distance from adjacent primary habitats and the permeability of a cropland matrix, have significant effects on local patterns of diversity[30][32]. Pairwise comparisons between oil palm and forest habitats could mask or underestimate differences in species richness if sampling effort is concentrated at fixed distances, especially near forest edges[33]. On the other hand, responses to the spatial configuration of agricultural mosaics can be highly variable among species functional groups[34]. Species attributes such as body mass, trophic level, home range size, dispersal capacity and degree of habitat specialization, define the ecological plasticity by which several species may or may not be able to tolerate severely modified habitats[35][36].

Both terrestrial and arboreal forest mammals can be severely affected by a broad spectrum of anthropogenic habitat disturbance in Amazonian forests[37][38]. However, the high diversity of phenotypes and ecological traits of different functional groups, reflect their diverse responses to environmental change[39]. In Peninsular Malaysia, terrestrial mammal species richness in oil palm monoculture was significantly reduced, compared to natural forest patches[40]. Vertebrates characterized by strict forest habitat affiliation, such as neotropical primates, are apparently most affected by forest conversion to cropland[41]. Wide-ranging species with vast spatial requirements, such as large carnivores, can occasionally use oil palm habitats near remaining forest patches[40]. Small carnivores, including small cats and civets, frequently use oil palm plantations in Sumatra, but their occupancy is affected by proximity to the forest edge[42]. Species reported to use oil palm landscape mosaics in Peninsular Malaysia, typically have generalist diets[40]. All available evidence from studies in Southeast Asia, therefore, indicates that both species traits and the grain and spatial configuration of oil palm monoculture affect their overall pattern of forest wildlife occupancy.

In Amazonia, areas most likely to be converted to oil palm plantations overlap the highest species richness of threatened birds and mammals[43]. There has been no attempt to examine the effects of primary and secondary forest conversion into oil palm monoculture on the Amazonian mammal fauna. Here, we investigate how midsized to large-bodied terrestrial and arboreal mammals respond to an Eastern Amazonian landscape mosaic, including oil palm plantations and large remnants of primary forest. These mammal taxa account for a disproportionate amount of the overall vertebrate biomass in Amazonian forests[44]. So any adverse effects to these species could amount to profound repercussions to ecosystem functioning across entire landscape mosaics. We compared different compartments of oil palm plantations with adjacent primary forest set-asides, using a standardized edge-distance gradient within each habitat. We describe habitat differences in species richness, overall abundance and species composition, and attempt to pinpoint key species life-history traits that govern species intolerance (or lack thereof) to oil palm monoculture. Finally we discuss the implications of these effects on biodiversity, considering the prospects for oil palm expansion in both aging and new Amazonian deforestation frontiers.

## Material and methods

### Study site

**Fig 1:**
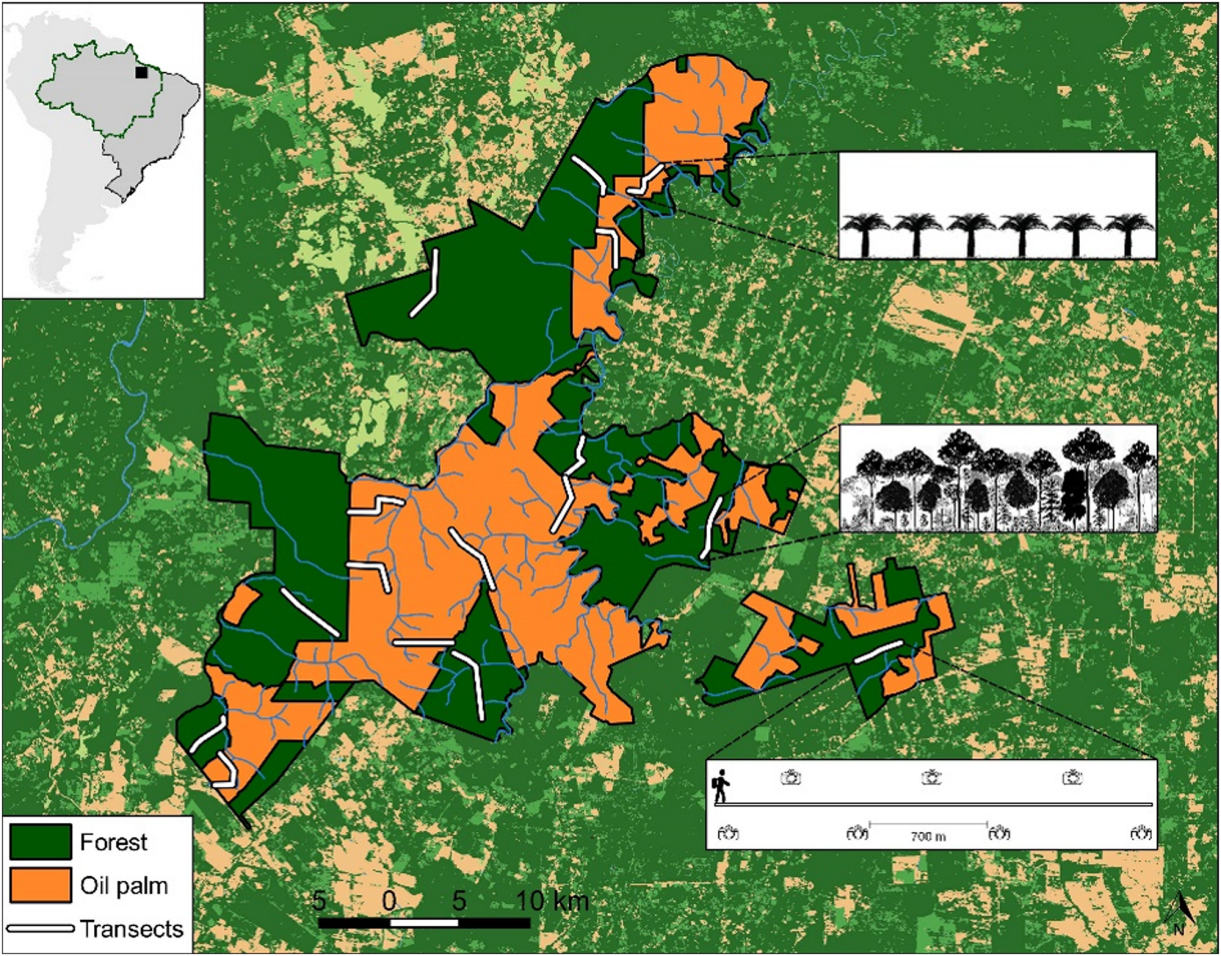
Location of the study area. Location of the study area in Eastern Brazilian Amazonia (solid square in inset map of South America). The main map represents the boundaries of the study area and the spatial distribution of 16 transects (white lines) in both habitat types, along which line-transect censuses and camera-trapping surveys were conducted. Dark green and orange polygons indicate primary forest and oil palm plantations, respectively, within the landscape mosaic. The diagram (lower right) provides details of the spatial configuration of camera-trapping stations along one of the transects. Inset figures show the typical structure and vertical profile of each habitat type: oil palm plantations (above) and primary forest (below). The background shows remaining forest cover, represented by different shades of green, and anthropogenic land cover (e.g., pasture and agriculture, shown in light brown) across the entire neighboring region. The map and satellite free source: MapBiomas Project [2017] Brazil's Annual Coverage and Land Use Map Series, acessed in [2017] link: [http://mapbiomas.org].

This study was conducted within the 103,000-hectare Agropalma private landholding (1°55’57” S, 48°45'49” W). The study area is located in an Eastern Amazonian landscape within the State of Pará, Brazil, which contains 39,000 ha of oil palm plantations and 64,000 ha of unflooded (*terra firme*) primary forest (Fig 1). Following a history of deforestation since the 1970s[45], remaining forest patches interspersed with oil palm plantations ranged from 1,500 to 15,000 ha (Fig 1). This region had been exploited mainly by conventional timber extraction and forest conversion into cattle pastures, but more recently a government-subsidized process of forest conversion into oil palm plantations. Most extensive oil palm plantations were consolidated since the 1980s, particularly in the municipal counties of Moju and Tailândia[46]. The broader landscape within the study region is currently a mosaic of anthropogenic open-habitat areas and natural forest remnants under varying degrees of degradation[47] (Fig 1).

*Terra Firme* forests in the study landscape, which are representative of the native forests in this eastern Amazonian region, were set-aside as Legal Reserves within the Agropalma landholding, as required by Brazilian law. Primary forest sites sampled here had not succumbed to understory fires but had been exposed to a history of small-scale selective logging, although this was discontinued at least 20 years prior to the study. Forest canopy heights are typically in the range of 25–35 m and dominant tree families included the Lecythidaceae, Sapotaceae, Burseraceae, Moraceae, Violaceae, and Leguminosae. Mean annual temperature is ~26.6°C, mean annual precipitation is ~2,500 mm, and soils throughout the study landscape are mainly highly weathered acidic oxisols.

### Mammal population surveys

We selected eight oil palm (OP) plantation sites of comparable ages in relation to overall habitat structure (7–15 years-old), which were paired with eight neighboring primary forest (PF) sites (Fig 1). Paired OP-PF sites were at least 4 km apart from one another, thereby maximizing the degree of spatial independence. Transects of 5 km in length were cut and marked every 50m in both all OP and PF sites surveyed. Within OP sites, we avoided placing any portion of transects within 500 m of the nearest remnants of riparian forest along perennial streams, which were also legally required forest set-asides, to control for any additional forest edge effects. Our pairwise design required that transects were placed in neighboring OP and PF sites, thereby creating a distance gradient into each of these two main habitat types, but given the spatial constraints of the study landscape, it was rarely possible to set up a continuous 10-km transect either side of the OP-PF habitat interface (Fig 1). Three of the eight PF transects were placed farther away from forest edges, thereby allowing us to sample sites at farther distances (5 ‒ 12 km) from the nearest oil palm plantations. The same was the case for two of the eight OP transects, which were far apart (0.5 ‒ 5.5 km) from the nearest areas of primary forest.

We conducted mammal surveys in both habitat types (OP and PF) using two methods: line transect censuses (LTC) by observers on foot and camera trapping (CT). A total of 627 km of transect census walks were carried out in April-May and October-November 2013, which included both the dry and wet seasons. Slow census walks (~1250 m h^–1^) were conducted by at least two independent observers from early in the morning (05:30h to 09:30h) and in the afternoon (15:30h to 19:30h) along alternate transects to match the typically bimodal activity rhythm of most forest vertebrates[48]. To maximize temporal independence, we never surveyed the same transect within less than a 4-day interval between consecutive census walks. In addition, a total sampling effort of 6,720 camera trap-nights was deployed from December 2014 to December 2015, which also included both the dry and wet seasons. We deployed seven CT stations per transect, each of which was spaced apart by approximately (but never less than) 700 m (Fig 1, S1 Fig), with a group of four paired transects (two in OP and two in PF) sampled simultaneously. This allowed us to camera-trap all 16 transects within 12 months. Each CT deployment was exposed for periods of 60 consecutive days, using high-capacity memory cards. Although we always attempted to deploy all cameras for 68 days, occasional malfunction and theft resulted in inconsistent deployment durations. When cameras were removed, a note was made of any problems or malfunctions such as water ingress, insect attack, dislodgement or battery failure. Mean functioning camera-trap night (FCTNs) per CT deployment was 54.61 ± 20.23. CT photographs were defined as an independent event if consecutive photos recorded (i) one or more individuals of different species; or (ii) one or more individuals of the same species over a minimum time interval greater than 60 min[49]. Using these criteria, all photos defined as non-independent were excluded from subsequent analyses.

### Habitat structure

We quantified the forest habitat structure at all mammal survey sites to understand how this may affect mammal species richness, composition and abundance. Along each transect, we placed 14 plots of 10 x 50 m in both habitats, seven of which on either side of each transect (S1 Fig), which amounted to a total of 224 plots. Within each plot, we measured all trees larger than 5 cm DBH (Diameter at Breast Height) and calculated the forest basal area (BA, in m^2^/ha) using the equation *BA* = *π*.*DBH*^2^/4. Oil palm trees within OP sites were excluded from this measure, as we aimed to restrict our sampling to native vegetation features.

In addition, using the QGis (v. 2.14) software, we measured the nearest distance from each sampling point to any perennial stream and to the nearest edge bordering the adjacent matrix (OP in the case of PF, or vice-versa). We also calculated the habitat patch area (ha) of all sampling sites, selecting shape polygons using the Field Calculator function in QGis. In addition to habitat type, we therefore also considered as environmental predictors of mammal community structure the (i) distance from each sampling point to the nearest OP-PF habitat interface (edge); (ii) basal area of native trees (ba); (iii) nearest distance to any permanent watercourse (stream); and (iv) forest patch area (area).

### Data analysis

We analysed the line-transect census (LTC) and camera-trapping (CT) data separately, as these two techniques target different components of the mammal fauna, some of which are mutually exclusive, with diurnal/arboreal and nocturnal/terrestrial vertebrates sampled primarily by LTC and CT, respectively. All LTC data analyses considered individual species records per 10-km of census walks, whereas CT data were treated as independent photographic records per 100 FCTNs. We first used Student´s paired t-tests to examine differences in total species richness, total numerical abundance, total biomass, and evenness values of the mammal fauna between habitat types on paired transects in either OP or PF, considering each survey technique separately. To standardize the differences in sample sizes (i.e. number of detection events in either LTC or CT), we estimated the species richness per transect based on abundance-based rarefaction curves using Chao 1 estimator, considering the lowest number of detections[50]. Evenness values were calculated as the *Pielou* index (J’), which was derived from the Shannon index, using the *Diversity* package within R. We selected this evenness measure because it is the most widely used in ecology, and is an excellent species abundance predictor of species richness in tropical forests[51]. J’ values range from 0.0 to 1.0, with higher values representing more even species distributions.

We examined the multivariate patterns of species composition in either OP or PF sites using Principal Coordinates Analysis (PCoA) based on species abundances, Bray-Curtis dissimilarity distances, and 1000 randomizations using the *vegan* R package. An analysis of species assemblage similarity between samples was then conducted using Permutational Multivariate Analysis of Variance (PERMANOVA), in which each transect was segmented at every 700-m. This allowed us to examine differences in mammal assemblage structure along transects as a function of local landscape context. We used Similarity Percentages Analysis (SIMPER) to break down the contribution of each species to the overall observed similarity between samples. We also calculated the mean (± SE) detection rate per transect to consider individual species responses to each habitat type. To assess the importance of habitat edge effects to differences in assemblage structure within each habitat type, we used Analysis of Covariance (ANCOVA). We compared the species composition and abundance across neighbouring habitats, using distances from the nearest edge (within either OP or PF) as a covariate.

To understand the effect of mammal life-history traits on patterns of species occupancy in either habitat type, we used Multiple Regression Matrices (MRM). This approach combines a response matrix, which in this case represented the species-by-site matrix weighed in terms of local abundance, with other dissimilarity matrices calculated from explanatory species data, including body mass, locomotion habit, degree of dietary specialization, phylogenetic distance, and degree of primary forest habitat dependence or specialization. These morpho-ecological traits, which included categories, ranks and continuous values, are described in S1 Table. To assess the relative importance of different environmental predictors on mammal species richness and abundance, considering the LTC and CT data separately, we applied Generalized Linear Mixed Models (GLMM) using the *glmmPQL* function within the *mass* R package. GLMMs were structured using a spatially hierarchical design, whereby census walks on transect segments or CT deployments were nested within transects, which are here defined a random variable. Environmental predictors (edge; ba; stream and area), defined above, were included in the GLMMs models.

## Results

**Fig 2:**
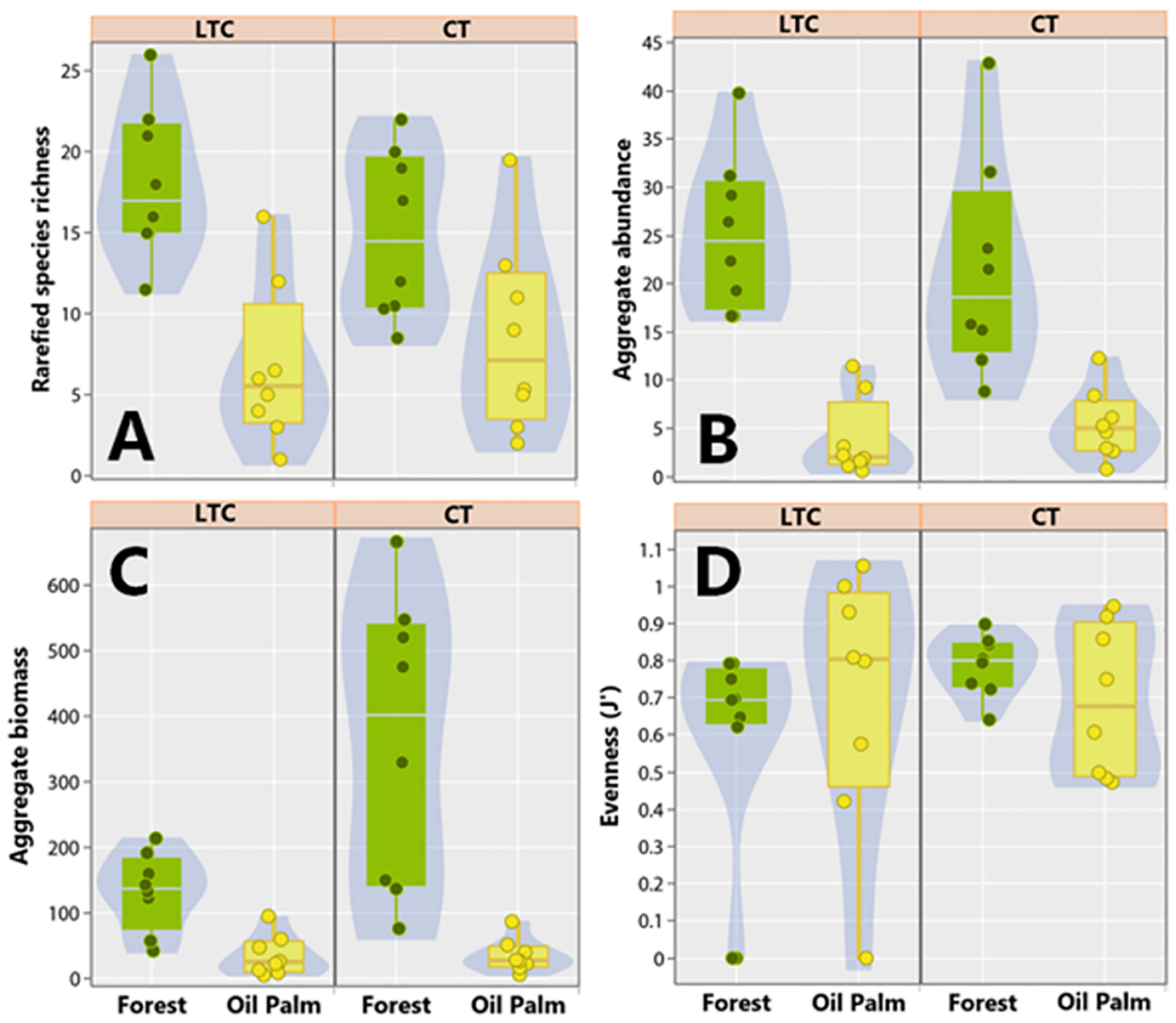
Comparison between oil palm plantations and forest considering overall patterns of mammal assemblage. Box and violin plots comparing the general patterns of mammal community structure between primary forest (in green) and oil palm plantations (in yellow), based on either line transect censuses (left panels) or camera trapping surveys (right panels). Four mammal assemblage properties were quantified: (A) Rarefied species richness; (B) Aggregate abundance; (C) Aggregate biomass; and (D) Community evenness.

Based on both line-transect censuses (LTC) and camera-trapping (CT), we recorded 1,059 observations of 36 medium and large-bodied terrestrial mammal species, including 310 sightings during LTC surveys and 749 independent photos from CT. A total of 32 and 23 species were recorded on the basis of either LTC or CT, respectively, in both primary forest and oil palm plantations (S2–S6 Figs and S1 Table). Considering data from both survey techniques, overall species richness and abundance were significant higher in primary forest compared to oil palm monoculture (Fig 2A and 2B; S2 Table). Aggregate biomass was also significantly greater in PF, compared to OP (Fig 2C and S2 Table). However, there were no differences in evenness estimates between the two habitat types (Fig 2D and S2 Table), suggesting similar relative abundance distributions of rare and common species.

**Fig 3:**
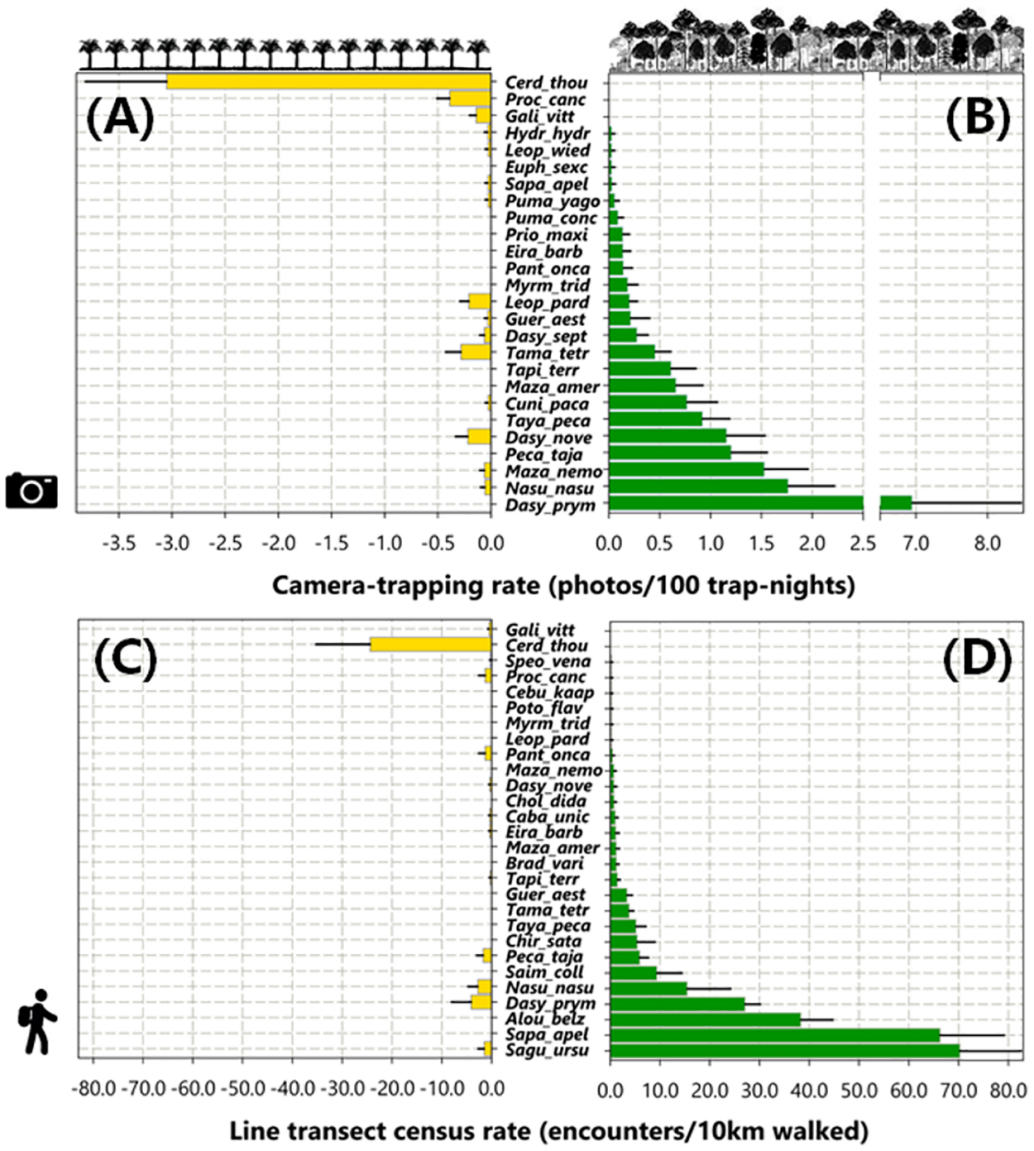
Relative abundance rates of terrestrial and arboreal mammal species observed in oil palm plantations and primary forest. Relative abundance rates in oil palm plantations (left panels: A, C) and primary forest (right panels: B, D) on the basis of camera trapping (upper panels: A, B) and line transect censuses (lower panels: C, D). Mammal species are represented by the first four letters of each genus and first four letters of each species, and ordered top to bottom in decreasing levels of abundance in primary forest.

Nocturnal terrestrial mammals (S1 Table) were most efficiently represented in CT samples (Fig 3A and 3B), whereas arboreal and scansorial species were far more frequently recorded during LTCs (Fig 3C and 3D). A total of 18 of all 36 mammal species recorded in this study were detected by both LTC and CT, and these survey techniques revealed similar patterns of relative abundance between PF and OP sites for 83% of those species (Fig 3).

**Fig 4:**
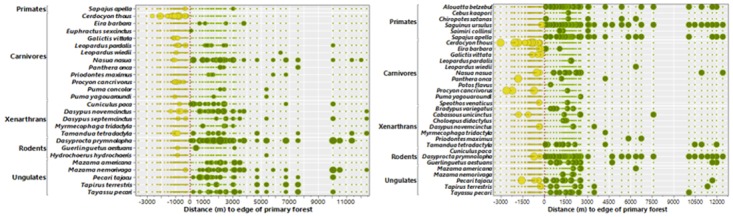
Relative abundance of terrestrial and arboreal mammal species along a distance gradient intersecting both oil palm plantations and primary forest. Distance gradient of oil palm plantations (yellow circles) and primary forest (green circles). Survey distances covered a gradient of up to 3500 m in oil palm and over 12,000 m in primary forest. Vertical red dashed line represents a 0-m distance along the edge interface between these two habitat types. Species are ordered according to their higher mammalian taxa (orders). Panels on the left (A) and right (B) represent data based on camera trapping and line transect censuses, respectively. Circle sizes are scaled according to log-transformed (ln x + 1) measures of local abundance based on either sampling technique. Very small dots represent non-detections at any given sampling point.

Virtually all arboreal species, including sloths (*Choloepus didactylus* and *Bradypus variegatus*), squirrels (*Guerlinguetus aestuans*), kinkajous (*Potos flavus*), and particularly primates, failed to be detected in oil palm plantations (Fig 3A and 3C). Considering primates, black-handed tamarins (*Saguinus ursulus*) and brown capuchin monkeys (*Sapajus apella*) were the only species observed using oil palm patches, but in all cases, they were detected within 300 m of primary forest (Fig 4). Considering xenarthrans, the two sloths, giant anteater (*Myrmecophaga tridactyla*) and giant armadillo (*Priodontes maximus*) were only recorded in primary forest (Fig 3), whereas three species of generalist armadillos (*Dasypus novemcinctus*, *Dasypus septemcinctus* and *Cabassous unicinctus*) were recorded in both habitats (Fig 3). Conversely, crab-eating foxes (*Cerdocyon thous*), which rarely use forests and are typical of more open habitats, were frequently and exclusively recorded in oil palm plantations. This was also the case of greater grison (*Galictis vittata*), but this carnivore species was rarely detected (Fig 3). The crab-eating raccoon (*Procyon cancrivorus*) was also more frequently detected at oil palm patches than at forest sites. These species, as well as jaguar (*Panthera onca*) and smaller cats (jaguarundis, *Puma yagouaroundi* and ocelots, *Leopardus pardalis*) clearly indicate that all terrestrial carnivores were either tolerant of or preferred oil palm monoculture. Most other species detected in both habitats were more abundant in primary forest, particularly ungulates (e.g. lowland tapir, *Tapirus terrestris*, grey brocket deer, *Mazama nemorivaga* and collared peccary, *Pecari tajacu*) and large-bodied rodents, such as pacas (*Cuniculus paca*) and agoutis (*Dasyprocta prymnolopha*) (Fig 3 and S1 Table).

**Fig 5:**
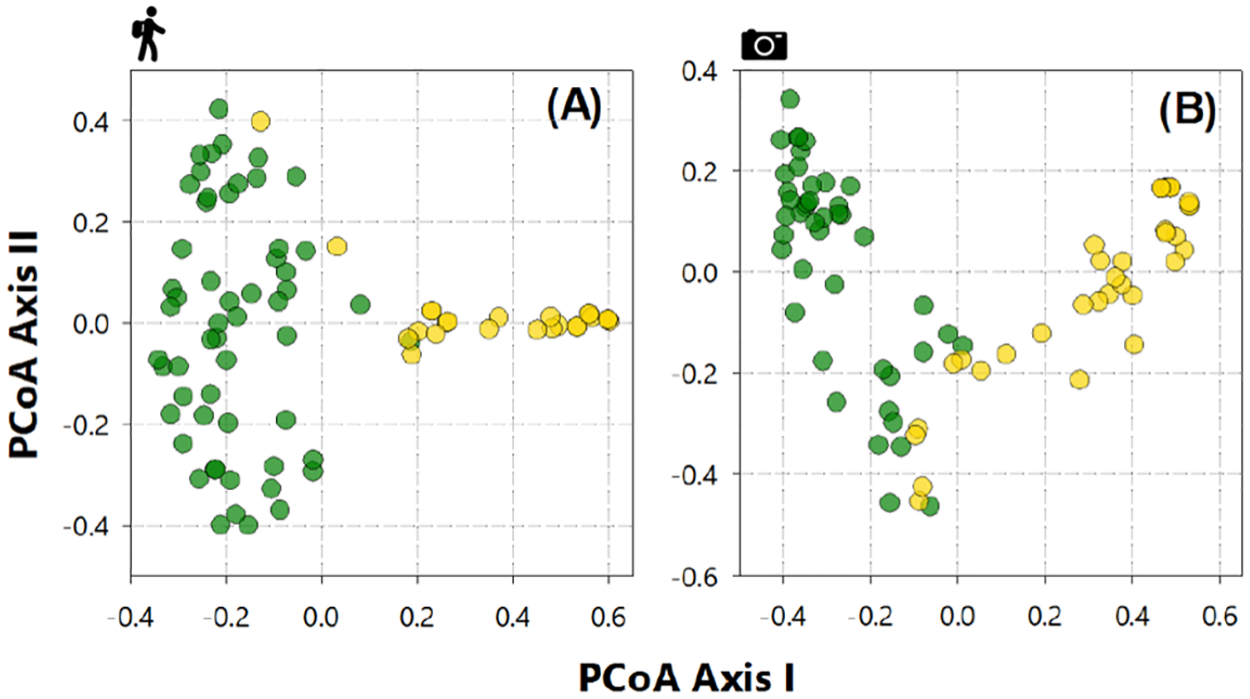
Principal Coordinates Analysis (PCoA) ordination of the mammal assemblage structure in primary forest and oil palm plantations across the study landscape. Mammal assemblage structure in primary forest (PF, green circles) and oil palm plantations (OP, yellow circles). PCoA plots are based on the dissimilarity matrix derived from the relative abundance data for each species based on either (A) line transect censuses or (B) camera trapping.

PCoA ordination showed clear differences between sample clusters within either primary forest or oil palm plantations, considering the species composition on the basis of both LTC (Fig 5A) and CT (Fig 5B), which was further confirmed by permutation tests (PERMANOVA; LTC: F_(80)_ = 19.84, p = 0.001; CT: F_(79)_ = 24.91, p = 0.001). However, PCoA clusters derived from the CT data show more overlap between samples in different habitats, suggesting that several terrestrial mammal species detected in PF also used OP patches (Fig 5B). SIMPER analysis further showed that on the basis of CT, two terrestrial species (agouti and crab-eating fox) had a significant contribution to the overall similarity between forest (23.0%) and oil palm samples (17.3%), respectively. Considering the LTC data, SIMPER analysis showed that crab-eating fox also had a significant contribution to the similarity between PF and OP samples (14.4%). However, three primate species—black-handed tamarin, brown capuchin monkey and red-handed howler monkey (*Alouatta belzebul*)—contributed with 11.8%, 9.1% and 8.4%, respectively, and jointly with agoutis (11.7%), were the main contributors to the overall similarity between PF samples (Fig 5A).

Distances to the nearest OP-PF edge interface had a significant effect on mammal assemblage structure within either habitat type considering both the LTC (Ancova; F_(2,81)_ = 41.77, p<0.001) and CT data (F_(2.77)_ = 19.97, p<0.001; Fig 5). A total of 20 of all 23 (87%) species recorded in oil palm plantations were never detected farther than 1300m from primary forest, and excluding the only truly non-forest species (crab-eating fox), median distances from the nearest forest edge for any mammal detected in oil palm was 960m for CT detections (*N* = 55 photos) and 927m for LTC detections (*N* = 47 sightings). Except for a few records of terrestrial carnivores far for the forest edge (>2000m), such as crab-eating raccoon and jaguar, only crab-eating foxes were detected at high abundance in oil palm (*N* = 34 sightings from LTC and *N* = 104 photos from CT; Fig 4).

On the basis of the first PCoA axis describing the mammal species composition, paired sites along the same or neighboring transects cutting across neighboring oil palm and primary forest sites were not necessarily less dissimilar than sites sampled along transects farther apart (ANOVA; LTC data: F_1,80_ = 282.6, *P* < 0.001; CT data: F_1,78_ = 266.2, *P* < 0.001). Transect identity had no effect (LTC data: *P* = 0.975; CT data: *P* = 0.601), indicating that habitat type was far more important than the spatial effects of transect identity in differentiating the community structure.

Multiple Regression Matrices analysis showed that species life-history traits had a significant effect on the overall pattern of species composition in both habitat types (R^2^ = 0.17, p = 0.001), and that this effect can be primarily attributed to species differences in the degree of forest habitat specificity (R^2^ = 0.04, p = 0.001) (S1 Table). This suggests that generalist or open countryside species that range widely into open habitat areas are tolerant of oil palm plantations, whereas both arboreal and terrestrial forest specialists are not. This reinforces the detrimental effect of oil palm habitat structure particularly on strictly arboreal species such as primates and sloths, for example. Conversely, habitat generalists that are widely known to use open areas, such as crab-eating fox, were clearly favored by oil palm plantations.

**Fig 6:**
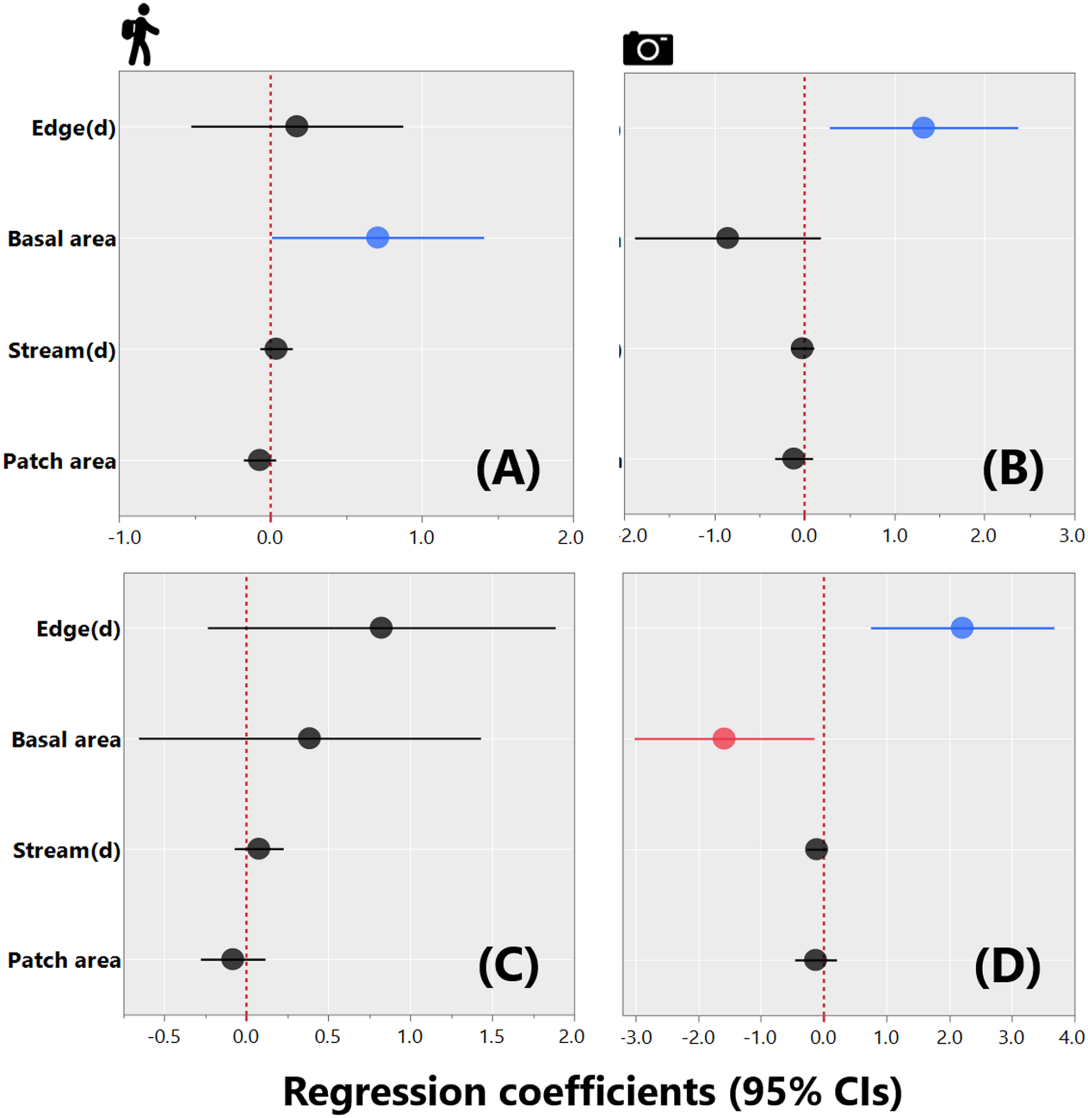
Coefficient estimates (± 95% confidence intervals) showing the magnitude and direction of effects of different explanatory variables. Effects of different explanatory variables considering either the line transect census data (A, C) or camera trapping data (B, D). Top panels (A, B) show effect sizes for species richness; bottom panels (C, D) show effect sizes for overall abundance. Explanatory variables included Edge_(d)_: distance to the nearest edge between primary forest and adjacent oil palm plantation; Basal area of native vegetation; Stream_(d)_: distance to the nearest perennial stream; and Patch area: size of any given habitat patch (in ha).

GLMMs revealed that forest basal area and distance to the nearest habitat interface were significant predictors of mammal species richness, considering the LTC and CT data, respectively (Fig 6A and 6B). On the other hand, considering the CT data alone, forest basal area was a significant negative predictor of overall abundance, whereas distance to the interface between OP and PF had the opposite effect (Fig 6D). Distance to neighboring streams and forest patch size failed to explain either mammal species richness or abundance (Fig 6).

## Discussion

We have shown that well-established oil palm plantations in Eastern Amazonia have clear detrimental effects on the assemblage of midsize to large-bodied mammals, and that some life-history traits were key determinants of species responses. Oil palm plantations were consistently impoverished compared to neighboring native forests, in terms of the general patterns of assemblage organization, including species richness, overall abundance and a measure of aggregate biomass across all species. Of all 23 terrestrial, 11 arboreal and two scansorial mammal species considered in this study, only three could be described as thriving in oil palm monoculture; all other species may use oil palm patches in their immediate forest neighborhood, but would likely be extirpated in the complete absence of primary forest within the wider landscape mosaic.

This general pattern is consistent with comparable results on the local avifauna surveyed within the same forest landscape, which showed that oil palm plantations were more impoverished in species composition than cattle pastures, secondary forests and primary forests within the same region[23]. In another study in natural savannas of the Colombian Llanos, species richness and abundance were severely reduced in areas converted into oil palm, and there were marked difference in species composition between habitats[24]. In contrast, a study in the Colombian Amazon showed that ants, dung beetles, and birds were more diverse in oil palm plantations than in pasture areas, and that oil palm could support a wide range of forest species[20]. However, studies using space-for-time substitution (i.e. those lacking “before and after” data), should pay close attention to the nature of baseline forest sites inferred as controls or pseudo-controls. In this study, the Agropalma study area has been historically embedded within an old Amazonian deforestation frontier, where virtually all remaining primary forest patches had been selectively logged in the past[45]. In addition, occasional hunting of forest vertebrates still occurs, despite the best efforts from the oil palm company to control access by hunters. Both timber extraction and hunting are expected to reduce the abundance and/or occupancy of several mammal species, thereby suggesting that assemblage-wide differences between oil palm and adjacent primary forest were somewhat underestimated here. Conversely, oil palm patches next to a more pristine forest baseline would have likely provided an even greater contrast in species habitat use.

Mammal species composition in oil palm was markedly different compared to adjacent primary forest. This was particularly the case of forest specialists, including most strictly arboreal species, such as primates and sloths, which were likely most affected by the lack of horizontal connectivity in the midstory and canopy layers, and severe simplification of habitat structure and the resource spectrum in oil palm plantations. Oil palm plantations also exhibit a much lower discontiguous canopy[18], which prevents most arboreal leapers and quadrupedal climbers from moving in horizontal space. Habitat use studies on neotropical primates, which are clearly more arboreal than their paleotropical counterparts of comparable size classes[52], show strong positive selection for complex forest habitats[53]. However, terrestrial forest specialists were also substantially affected by oil palm plantations, as the habitat structure of this oil-seed crop at the ground layer is also in marked contrast to that of native forest. This includes elevated soil exposure, reduced leaf litter[15][16], lack of dead-wood substrates, and lack of an understory[17], rendering several ungulates (e.g. *Mazama americana* and *Tayassu pecari*), large rodents (*Cuniculus paca* and *Dasyprocta prymnolopha*), and xenarthrans (e.g. *Priodontes maximus* and *Myrmecophaga tridactyla*) conspicuously absent from oil palm. Even the high productivity and nearly year-round availability of oil palm fruits apparently fails to compensate for the severe structural differences between this tree monoculture and a diverse forest habitat.

The lower overlap in ordination (PCoA) space between LTC samples in different habitats clearly indicates higher dissimilarity between site clusters in either OP or PF, again reinforcing the notion that arboreal mammals, which were sampled almost exclusively by census walks, were more affected by oil palm plantations. Despite differences in species selectivity between camera-trapping and line-transect censuses, results based on either one of these methods were still generally consistent in relation to overall community patterns.

We observed a large lateral spill-over effect in animal populations from primary forest to adjacent oil palm plantations, which was clearly represented by several ungulate and large rodent species, but particularly mid-sized carnivores. The general use of oil palm plantations by wide-ranging carnivores is likely driven by the higher prey biomass density for at least some apex predator species. In a parallel 1-year long study at the same landscape, we conducted a systematic live-trapping effort to sample small mammals (rodents and marsupials) in both vegetation types (ACMO et al., unpubl. data). We uncovered a very high density of rodents in oil palm monoculture (65% of the total abundance including both oil palm and primary forest sites), which included particularly common species, such as *Hylaeamys megacephalus* and four species of *Oecomys*. These insectivore/frugivore rodents[54] are probably attracted by the nearly year-round high yield of oil palm fruits, but also by the high abundance of arthropods present along the parallel rows of residual vegetation necromass generated by previous plantation cycles. This prey base likely attracted small cats, such as ocelots, jaguarundis and margays (*Leopardus wiedii*), but also other small carnivores, including mustelids (greater grison and tayra, *Eira barbara*) and a forest canid (bush dog, *Speothos venaticus*).

This spill-over effect can be considered unidirectional from a natural forest to an entirely anthropogenic and intensively managed habitat[55]. Except for crab-eating raccoons, the only other two species that were more abundant within oil palm were not detected in adjacent forest patches. Of these, the greater grison is naturally rare[56], and although it may be considered a forest affiliate, this species is a habitat-generalist that is highly tolerant of disturbed areas[57]. The only species that we can categorically interpret as clearly favored by oil palm plantations is the crab-eating fox. This Brazilian *Cerrado* species specializes in open habitat areas[54], and has expanded its range and vastly increased its overall abundance throughout many anthropogenically disturbed parts of the Amazon[58].

A key discussion in the literature, which is partly motivated by bilateral agreements regulated by the Roundtable on Sustainable Palm Oil (RSPO), has focussed on plantation management standards that enhances native biodiversity retention within oil palm plantations[12][27][33]. In our study landscape, protecting large areas of natural forest remnants could clearly minimize the negative landscape-wide effects of oil palm plantations, compared to protecting small forest fragments. Large protected areas support larger populations that can operate as source[59][60]. Many of these populations can take advantage of ecological subsidies from neighboring anthropogenic habitats via small-scale spill-over, but are unlikely to meet their metabolic requirements without sufficiently large forest areas. Although the spatial configuration of forest fragments around a nuclear oil palm-dominated matrix could enhance vertebrate dispersal across large cropland areas, this was only partly validated based on our observations since lateral movements by virtually all mammal species never exceeded 1300 meters into oil palm plantations. Distance thresholds from forest remnants of meaningful size are therefore clearly important in defining mammal responses to oil palm plantations[30][33]. In this study, local mammal diversity was rapidly reduced along increasingly greater distances from primary forest. This is because spill-over effects were largely restricted to the immediate forest neighborhood and most core areas of oil palm were rarely if ever used by most species. Furthermore, native riparian forests along perennial streams, which were set aside within areas of oil palm plantations, could act as corridors for wildlife if they could remain largely intact[61], but the environmental heterogeneity of these riparian corridors was severely reduced in oil palm areas, affecting even the aquatic biota[62]. In any case, several species that can use oil palm plantations, such as crab-eating raccoon and jaguarundi, can still benefit from degraded forest strips, as these species often use or disperse through riparian habitat[57]. The severe structural and compositional simplification in habitat heterogeneity associated with oil palm plantations is closely linked to the loss of vertical stratification, absence of an understory, and severe loss in forest basal area, all of which can help explain the depauperate mammal assemblages observed in oil palm.

We identified at least three “Vulnerable” mammal species according to the IUCN Red List, which were never recorded in oil palm plantations (*Priodontes maximus*, *Myrmecophaga tridactyla* and *Tayassu pecari*), in addition to two Critically Endangered primates (*Cebus kaapori* and *Chiropotes satanas*) which rarely used plantations. Considering the prospect of oil palm expansion in the Brazilian Amazonia[8][9], retaining large areas of primary forest within the plantation matrix is, therefore, the only option of guarding the conservation interests of these threatened species.

The Brazilian federal government enacted policies encouraging more benign forms of oil palm production in the Amazon by banning low-interest investment loans granted to those companies or smallholders who are likely to convert primary forest. However, more recent state-level legislation has been sanctioned to regulate oil palm plantations in forest areas (including secondary and logged primary forest) or on fallow land (e.g. *Instrução Normativa* SEMAS/PA/2011). Environmental licensing applications to convert young secondary forests into oil palm plantations can now be rubber-stamped as these areas are often considered highly degraded and of little value in terms of their natural capital. Yet most of the remaining forest area in Eastern Amazonia (~83%) is now comprised of secondary forests[47], most of which are threatened by further forest conversion into more lucrative land uses.

In 2011, the state government of Pará revoked a law that defined oil palm plantations as a reforestation option (Portaria SEMA/Pará 3872/2010), thereby signaling that this crop could be accounted for in the calculation of the 80% mandatory Legal Forest Reserve set-aside within private landholdings across the Amazon (Brazilian Federal Law No. 12.651/2012). However, the governance scenario in protecting native forests and biodiversity in private lands does not bode well given the current direct and indirect policy incentives for oil palm expansion in both old and new Amazonian deforestation frontiers, including facilitated rural credit for landowners and government investments in ancillary infrastructure such as roads and power lines.

In summary, Amazonian forest mammal responses to oil palm plantations are modulated by (1) severe differences in habitat structure and composition between species-rich primary forests and species-poor conventional oil palm plantations; (2) the influence of functional traits on individual species responses to novel habitat conditions; and (3) the effects of landscape structure, as well as the size, spatial arrangement and integrity of forest remnants in relation to the dominant matrix of oil palm. All these factors contributed to the drastic erosion of the mammalian fauna in industrial scale oil palm plantations in Eastern Amazonia. Our study therefore clearly reinforces the notion that oil palm plantations can be extremely hostile to native tropical forest biodiversity, as has been shown in more traditional oil palm countries in South-East Asia, such as Malaysia and Indonesia[12][21]. Our results paint a pessimistic scenario considering the extremely high suitability of most Amazonian soils and climatic conditions for oil palm monoculture and the rapidly growing demand for biofuels and vegetable oils around the world[8][9]. We therefore strongly recommend caution in sanctioning future direct or indirect government subsidies for the conversion of forest areas into oil palm plantations.

## Supporting information

S1 Fig Schematic sampling design used during mammal surveys and variables quantified. Example of a transect (A) that were approximately 5,000 m in length, along which line-transect mammal survey were conducted; The seven plots (B) where camera-trapping surveys were conducted, which were spaced apart by 700 m; and the floristic plots (B) and (C), measuring 10m x 50m, where native tree basal area was measured.
(DOCX)

S2 Fig Percentage of mammal records (pie charts in the upper corners) of semi-fossorial species, including A–*Priodontes maximus*, B–*Cabassous unicinctus*, C–*Dasypus novemcinctus*, D–*Dasypus septemcinctus*, E–*Euphractus sexcinctus*; arboreal species including, F–*Bradypus variegatus*, G–*Choloepus didactylus* and scansorial species including, H–*Tamandua tetradactyla*, sampled in oil palm plantation (orange pie chart) and primary forest (green pie chart), using both sampling methods: Camera Traps (inset camera) and Line-Transect census (inset observer on foot). Photos authors: A–Leonardo Maffei, available at https://www.researchgate.net/publication/269709095; D–Teresa Anacleto, available at http://dx.doi.org/10.2305/IUCN.UK.2014-1.RLTS.T6293A47441509.en; F–Adriano Chiarello, available at http://www.iucnredlist.org/details/3038/0; G - https://commons.wikimedia.org/wiki.
(DOCX)

S3 Fig Percentage of mammal records (pie charts in the upper corners) of terrestrial species, including A–*Myrmecophaga tridactyla* and B–*Galictis vittata* and arboreal species, including Primates: C–*Cebus kaapori*, D–*Saguinus ursulus*, E–*Chiropotes satanas*; F–*Alouatta belzebul*, G–*Saimiri collinsi* and H–*Sapajus apella*, sampled in oil palm plantation (orange pie chart) and primary forest (green pie chart), using both sampling methods: Camera Traps (inset camera) and Line Transect census (inset observer on foot). Photos authors: C–Liza Veiga, available at http://www.icmbio.gov.br/portal; F– https://en.wikipedia.org/wiki; G–José de Souza Junior, available at http://www.icmbio.gov.br/portal.
(DOCX)

S4 Fig Percentage of mammal records (pie charts in the upper corners) of arboreal species, including A–*Potos flavus*, scansorial species, including B–*Procyon cancrivorus* and C–*Nasua nasua* and terrestrial species, including, D–*Tapirus terrestris*, E–*Pecari tajacu*, F–*Tayassu pecari*, G–*Mazama americana* and H–*Mazama nemorivaga* sampled in oil palm plantation (orange pie chart) and primary forest (green pie chart), using both sampling methods: Camera Traps (camera figure) and Line-Transect census (observer on foot). Photo author: A–Katia Yoza.
(DOCX)

S5 Fig Percentage of mammal records (pie charts in the upper corners) of terrestrial species, including A–*Panthera onca*, B–*Puma concolor*, C–*Leopardus pardalis*, D–*Puma yagouaroundi*, E–*Speothos venaticus*, F–*Cerdocyon thous*, G–*Eira barbara*, and H–*Leopardus wiedii* sampled in oil palm plantation (orange pie chart) and primary forest (green pie chart), using both sampling methods: Camera Traps (camera figure) and Line-Transect census (observer on foot). Photo: H-http://procarnivoros.org.br/index.php/animais/gato-maracaja-leopardus-wiedii/.
(DOCX)

S6 Fig Percentage of mammal records (pie charts in the upper corners) of terrestrial species, including A–*Dasyprocta prymnolopha*, B–*Cuniculus paca*, C–*Hydrochoerus hydrochaeris* and arboreal species, D–*Guerlinguetus aestuans* sampled in oil palm plantation (orange pie chart) and primary forest (green pie chart), using both sampling methods: Camera Traps (camera figure) and Line Transect census (observer on foot). (DOCX)

S1 Table Species recorded at oil palm plantations and in primary forest. Species abundance in each habitat (oil palm plantations and primary forest), considering the rate of Camera Trapping records (CT) as the number of independent photographic records per 100 functioning camera-trap nights, and detection records during Line Transect (LT) census, as the number of individual species records per 10 km of census walked; Values and categories of functional traits used in the Multiple Regression Matrices analysis; and conservation status of each species recorded, as classified by the International Union for Conservation of Nature (IUCN).
(DOCX)

S2 Table Statistical results of student´s paired t-tests to examine differences in total species Richness, total numerical Abundance, total Biomass, and Evenness values of the mammal assemblages across habitat types on paired transects in either oil palm or primary forest. The results include degrees of freedom (df); t-value (*t*); and *p*-value (*p*).
(DOCX)
