# Supplementary material for: Oil palm monoculture induces drastic erosion of an Amazonian forest mammal fauna
Source: PLoS One. 2017 Nov 8;12(11):e0187650. doi: 10.1371/journal.pone.0187650 (PMC5695600; doi:10.1371/journal.pone.0187650)

**Supporting Information**

**Oil palm monoculture induces drastic erosion of an Amazonian forest mammal fauna**

Ana Cristina Mendes-Oliveira, Carlos A. Peres, Paula Cristina R. de A. Maués, Geovana Linhares Oliveira, Ivo G. B. Mineiro, Susanne L. Silva de Maria and Renata C. S. Lima

**S1 Figure. Schematic sampling design used during mammal surveys and variables quantified**. Example of a transect (A) that were approximately 5,000 m in length, along which line-transect mammal survey were conducted; The seven plots (B) where camera-trapping surveys were conducted, which were spaced apart by 700 m; and the floristic plots (B) and (C), measuring 10m x 50m, where native tree basal area was measured.


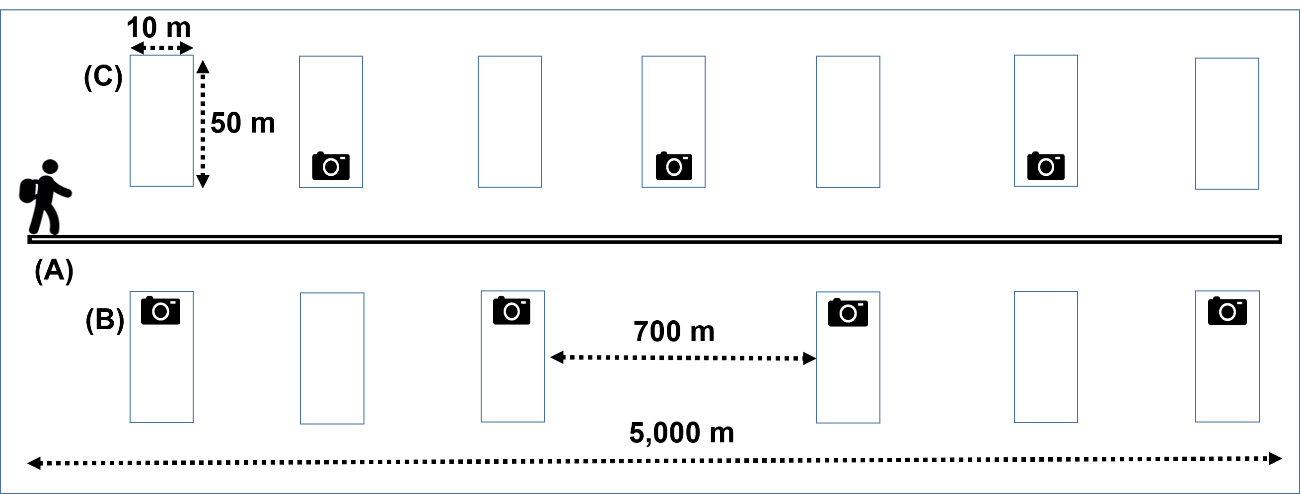

Supplement: S1 Fig — Example of a transect (A) that were approximately 5,000 m in length, along which line-transect mammal survey were conducted; The seven plots (B) where camera-trapping surveys were conducted, which were spaced apart by 700 m; and the floristic plots (B) and (C), measuring 10m x 50m, where native tree basal area was measured. (DOCX) [file pone.0187650.s001.docx]
