# Supplementary material for: Oil palm monoculture induces drastic erosion of an Amazonian forest mammal fauna
Source: PLoS One. 2017 Nov 8;12(11):e0187650. doi: 10.1371/journal.pone.0187650 (PMC5695600; doi:10.1371/journal.pone.0187650)

**S2 Figure.** Percentage of mammal records (pie charts in the upper corners) of semi-fossorial species including, A – *Priodontes maximus*, B – *Cabassous unicinctus*, C – *Dasypus novemcinctus*, D – *Dasypus septemcinctus*, E – *Euphractus sexcinctus*; arboreal species including, F – *Bradypus variegatus*, G – *Choloepus didactylus* and scansorial species including, H – *Tamandua tetradactyla*, sampled in oil palm plantation (orange pie chart) and primary forest (green pie chart), using both sampling methods: Camera Traps (inset camera) and Line Transect census (inset observer on foot). Photos authors: A – Leonardo Maffei, available at <https://www.researchgate.net/publication/269709095>; D – Teresa Anacleto, available at <http://dx.doi.org/10.2305/IUCN.UK.2014-1.RLTS.T6293A47441509.en>; F – Adriano Chiarello, available at <http://www.iucnredlist.org/details/3038/0>; G - <https://commons.wikimedia.org/wiki>


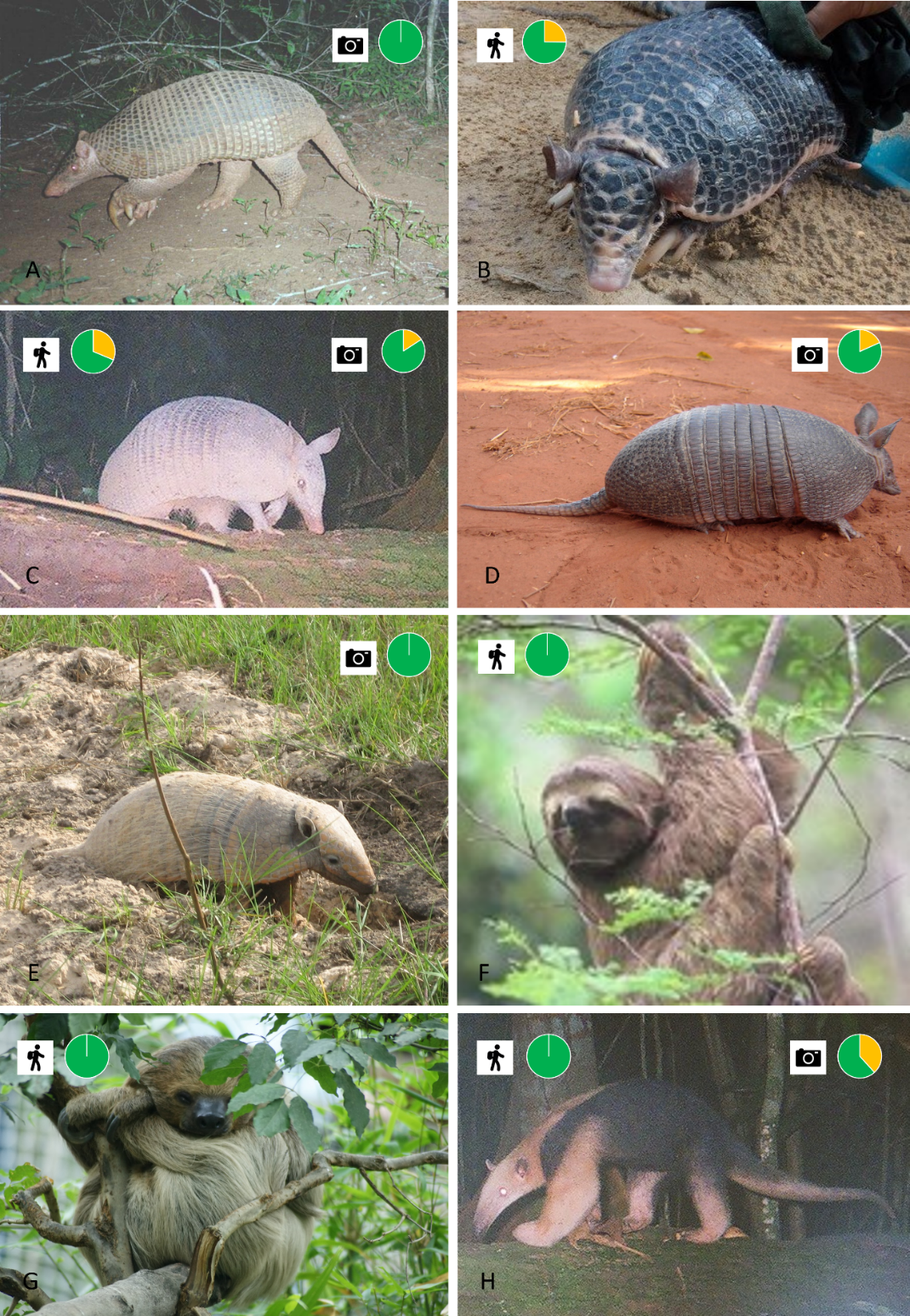

Supplement: S2 Fig — Photos authors: A–Leonardo Maffei, available at https://www.researchgate.net/publication/269709095; D–Teresa Anacleto, available at http://dx.doi.org/10.2305/IUCN.UK.2014-1.RLTS.T6293A47441509.en; F–Adriano Chiarello, available at http://www.iucnredlist.org/details/3038/0; G - https://commons.wikimedia.org/wiki. (DOCX) [file pone.0187650.s002.docx]
