# Supplementary material for: Oil palm monoculture induces drastic erosion of an Amazonian forest mammal fauna
Source: PLoS One. 2017 Nov 8;12(11):e0187650. doi: 10.1371/journal.pone.0187650 (PMC5695600; doi:10.1371/journal.pone.0187650)

**S3 Figure.** Percentage of mammal records (pie charts in the upper corners) of terrestrial species, including A – *Myrmecophaga tridactyla* and B – *Galictis vittata* and arboreal species, including Primates: C – *Cebus kaapori*, D – *Saguinus ursulus*, E – *Chiropotes satanas*; F – *Alouatta belzebul*, G – *Saimiri collinsi* and H – *Sapajus apella*, sampled in oil palm plantation (orange pie chart) and primary forest (green pie chart), using both sampling methods: Camera Traps (inset camera) and Line Transect census (insetobserver on foot). Photos authors: C – Liza Veiga, available at  [http://www.icmbio.gov.br/portal](https://www.researchgate.net/publication/269709095); F – <https://en.wikipedia.org/wiki>; G – José de Souza Junior, available at  [http://www.icmbio.gov.br/portal](https://www.researchgate.net/publication/269709095)


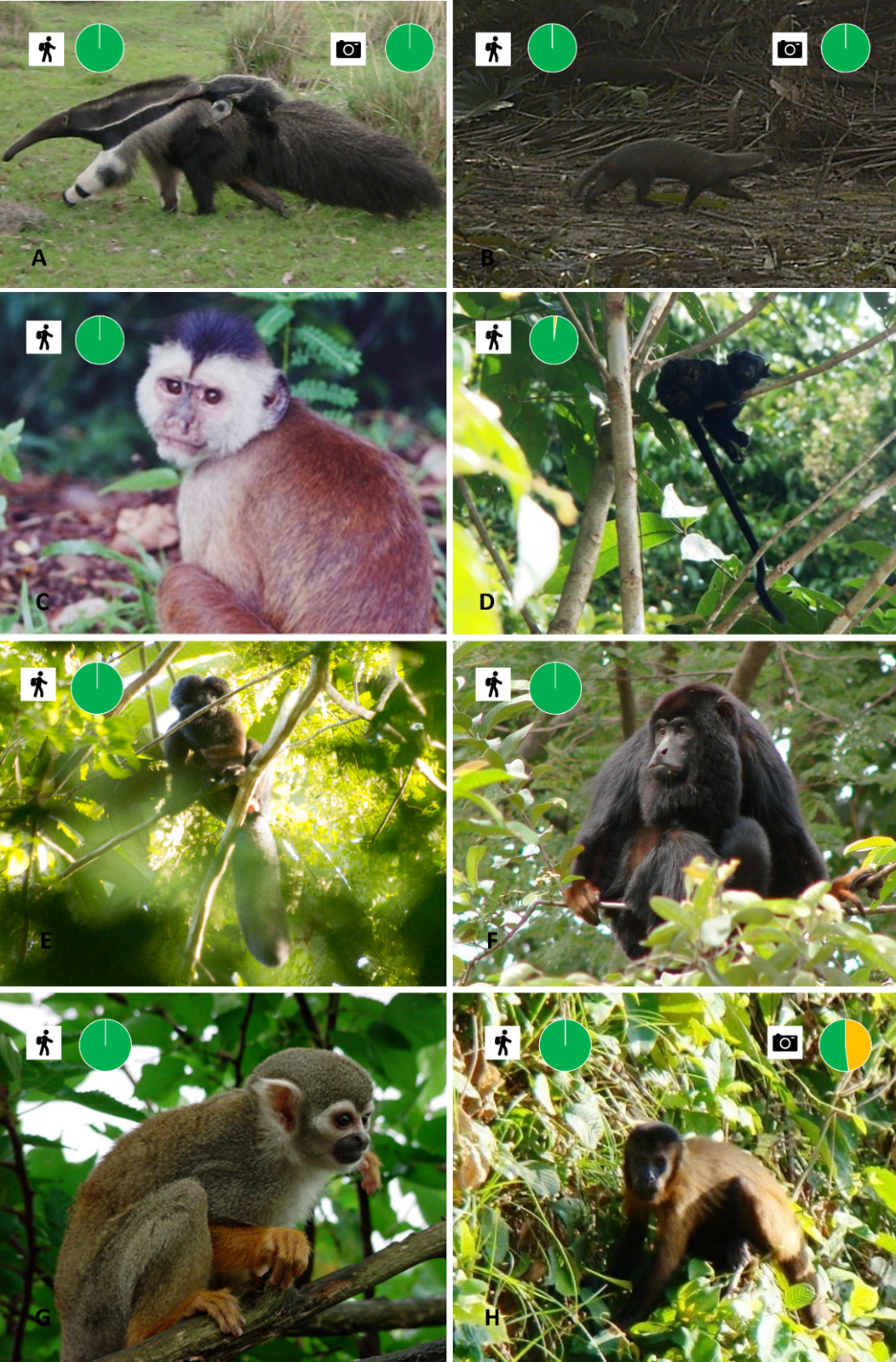

Supplement: S3 Fig — Photos authors: C–Liza Veiga, available at http://www.icmbio.gov.br/portal; F– https://en.wikipedia.org/wiki; G–José de Souza Junior, available at http://www.icmbio.gov.br/portal. (DOCX) [file pone.0187650.s003.docx]
