# Supplementary material for: Oil palm monoculture induces drastic erosion of an Amazonian forest mammal fauna
Source: PLoS One. 2017 Nov 8;12(11):e0187650. doi: 10.1371/journal.pone.0187650 (PMC5695600; doi:10.1371/journal.pone.0187650)

**S4. Figure.** Percentage of mammal records (pie charts in the upper corners) of arboreal species, including A – *Potos flavus*,, scansorial species, including B – *Procyon cancrivorus* and C – *Nasua nasua* and terrestrial species, including D – *Tapirus terrestris*, E – *Pecari tajacu*, F – *Tayassu pecari*, G – *Mazama americana* and H – *Mazama nemorivaga* sampled in oil palm plantation (orange pie chart) and primary forest (green pie chart), using both sampling methods: Camera Traps (camera figure) and Line Transect census (observer on foot). Photo author: A – Katia Yoza


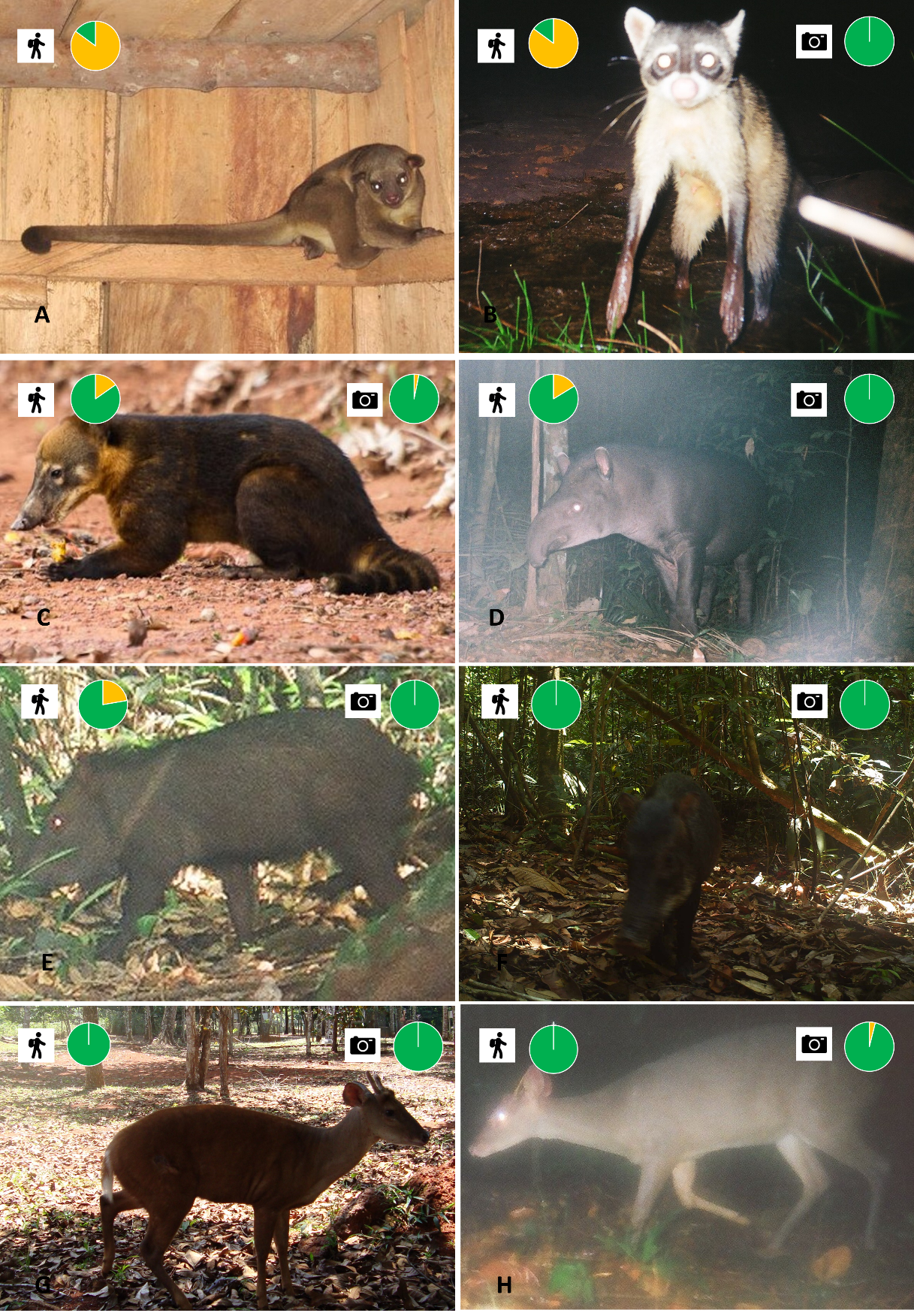

Supplement: S4 Fig — Photo author: A–Katia Yoza. (DOCX) [file pone.0187650.s004.docx]
