# Supplementary material for: Oil palm monoculture induces drastic erosion of an Amazonian forest mammal fauna
Source: PLoS One. 2017 Nov 8;12(11):e0187650. doi: 10.1371/journal.pone.0187650 (PMC5695600; doi:10.1371/journal.pone.0187650)

**S5 Figure.** Percentage of mammal records (pie charts in the upper corners) of terrestrial species, including A – *Panthera onca*, B – *Puma concolor*, C – *Leopardus pardalis*, D – *Puma yagouaroundi*, E – *Speothos venaticus*, F – *Cerdocyon thous*, G – *Eira barbara* and H – *Leopardus wiedii* sampled in oil palm plantation (orange pie chart) and primary forest (green pie chart), using both sampling methods: Camera Traps (camera figure) and Line Transect census (observed on foot). Photo: H- [http://procarnivoros.org.br/index.php/animais/gato-maracaja-leopardus-wiedii/](http://procarnivoros.org.br/index.php/animais/gato-maracaja-leopardus-wiedii/)


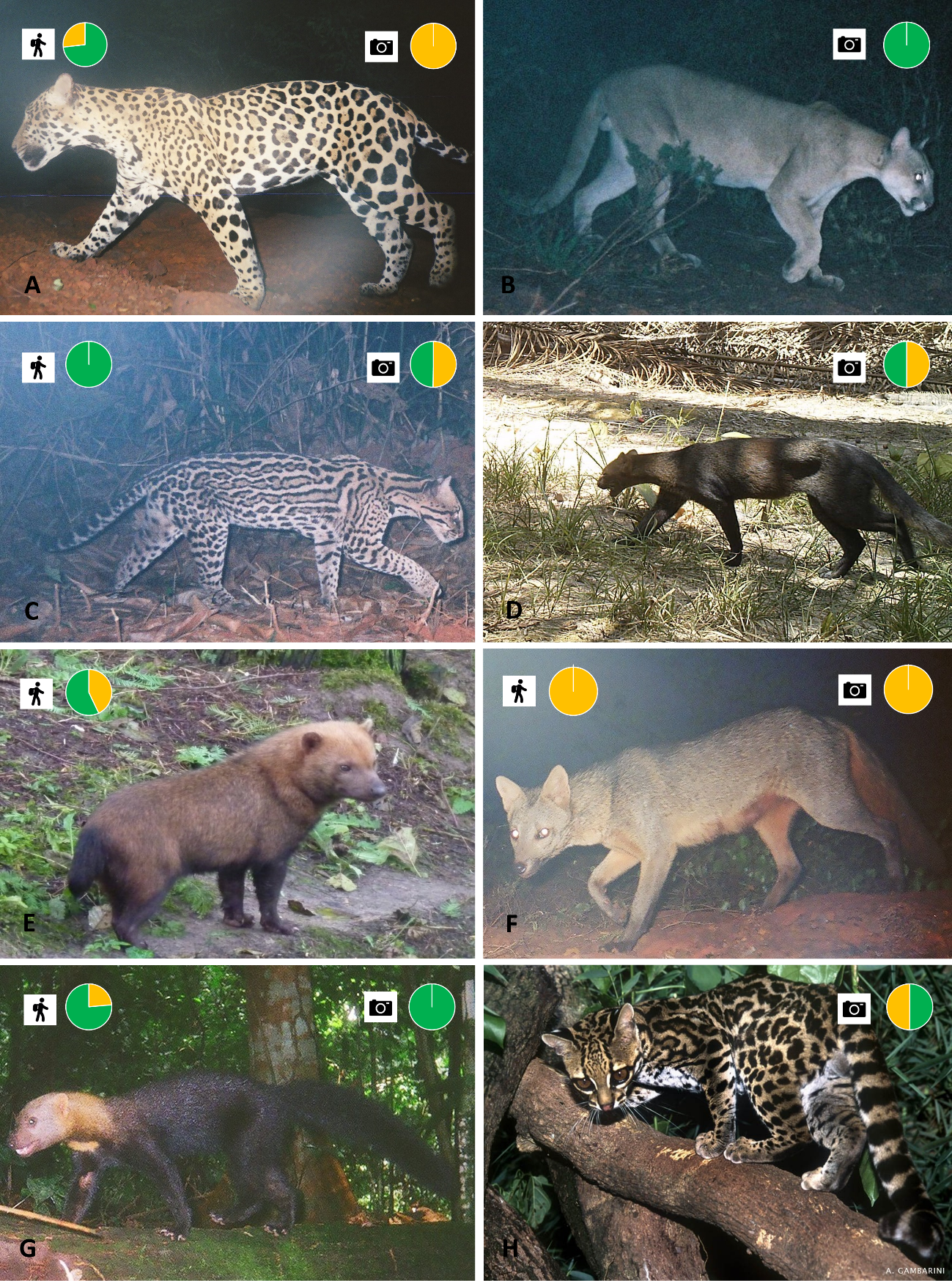

Supplement: S5 Fig — Photo: H-http://procarnivoros.org.br/index.php/animais/gato-maracaja-leopardus-wiedii/. (DOCX) [file pone.0187650.s005.docx]
