# Supplementary material for: Oil palm monoculture induces drastic erosion of an Amazonian forest mammal fauna
Source: PLoS One. 2017 Nov 8;12(11):e0187650. doi: 10.1371/journal.pone.0187650 (PMC5695600; doi:10.1371/journal.pone.0187650)

**S6 Figure.** Percentage of mammal records (pie charts in the upper corners) of terrestrial species, including A – *Dasyprocta prymnolopha*, B – *Cuniculus paca*, C – *Hydrochoerus hydrochaeris* and arboreal species, including D – *Guerlinguetus aestuans* sampled in oil palm plantation (orange pie chart) and primary forest (green pie chart), using both sampling methods: Camera Traps (camera figure) and Line Transect census (observer on foot).


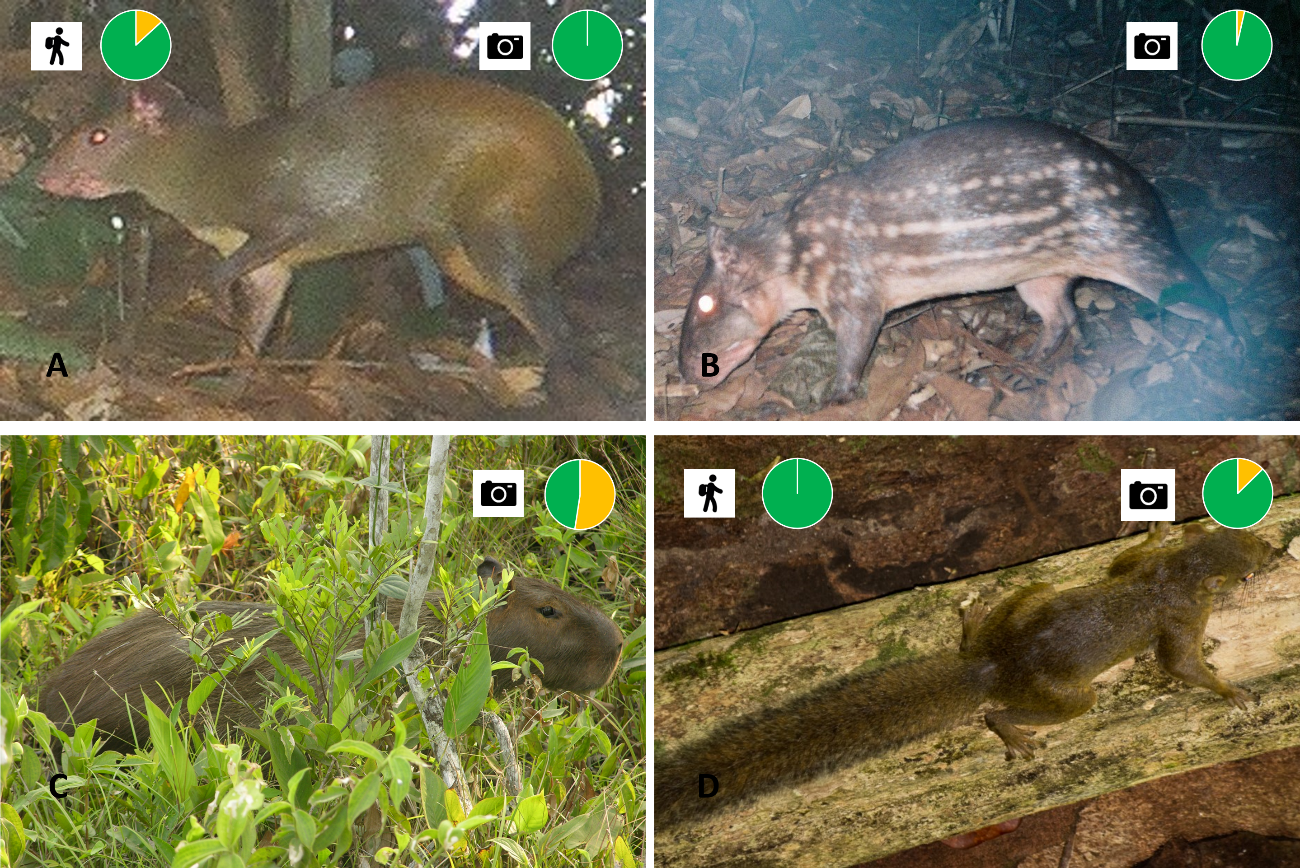

Supplement: S6 Fig — (DOCX) [file pone.0187650.s006.docx]
