# Supplementary material for: Oil palm monoculture induces drastic erosion of an Amazonian forest mammal fauna
Source: PLoS One. 2017 Nov 8;12(11):e0187650. doi: 10.1371/journal.pone.0187650 (PMC5695600; doi:10.1371/journal.pone.0187650)
Supplement: S1 Table — Species abundance in each habitat (oil palm plantations and primary forest), considering the rate of Camera Trapping records (CT) as the number of independent photographic records per 100 functioning camera-trap nights, and detection records during Line Transect (LT) census, as the number of individual species records per 10 km of census walked; Values and categories of functional traits used in the Multiple Regression Matrices analysis; and conservation status of each species recorded, as classified by the International Union for Conservation of Nature (IUCN). (DOCX) [file pone.0187650.s007.docx]

**S1 Table. Species registered in oil palm plantation and in the forest**. Species abundance in each habitat (oil palm plantations and primary forest), considering the rate of Camera Trapping records (CT) as number of independent photographic records per 100 functioning camera-trap night, and detection records during Line Transect (LT) census as the number of individual species records per 10-km of census walked; Values and categories of functional traits used in the Multiple Regression Matrices analysis; and conservation status of each species recorded, as classified by the International Union for Conservation of Nature (IUCN)

| Taxon | | Oil Palm | | Forest | | Functional traits | | | Conservation status  (IUCN) |
| --- | --- | --- | --- | --- | --- | --- | --- | --- | --- |
| Order | Species | CT | LTC | CT | LTC | Biomass (Kg) | Locomotion | Diet/Habitat Specialization* |  |
| Cingulata | *Dasypus novemcinctus* | 12.19 | 19.28 | 64.89 | 42.30 | 4.10 | Semifossorial | Generalist/Forest | Least Concern |
|  | *Dasypus septemcinctus* | 3.50 | - | 15.62 | - | 1.50 | Semifossorial | Generalist/Open area | Least Concern |
|  | *Euphractus sexcinctus* | 0 | - | 1.61 | - | 4.85 | Semifossorial | Generalist/Open area | Least Concern |
|  | *Priodontes maximus* | 0 | - | 7.87 | - | 40.00 | Semifossorial | Specialist/Forest | Vulnerable |
|  | *Cabassous unicinctus* | - | 19.28 | - | 57.26 | 4.2 | Semifossorial | Generalist /Forest | Least Concern |
| Pilosa | *Bradypus variegatus* | - | 0 | - | 69.01 | 3.90 | Arboreal | Specialist/Forest | Least Concern |
|  | *Choloepus didactylus* | - | 0 | - | 42.92 | 6.30 | Arboreal | Specialist/Forest | Least Concern |
|  | *Myrmecophaga tridactyla* | 0 | 0 | 10.52 | 13.89 | 30.50 | Terrestrial | Specialist/Forest | Vulnerable |
|  | *Tamandua tetradactyla* | 15.88 | 0 | 25.58 | 214.41 | 5.00 | Scansorial | Specialist/Forest | Least Concern |
| Primates | *Cebus kaapori* | - | 0 | - | 12.82 | 2.15 | Arboreal | Specialist/Forest | Critically Endangered |
|  | *Sapajus apella* | 1.61 | 0 | 1.75 | 3712.45 | 2.72 | Arboreal | Generalist/Forest | Least Concern |
|  | *Saimiri collinsi* | - | 0 | - | 525.21 | 0.76 | Arboreal | Generalist/Forest | Least Concern |
|  | *Saguinus* *ursulus* | - | 83.69 | - | 3935.61 | 0.39 | Arboreal | Generalist/Forest | - |
|  | *Chiropotes satanas* | - | 0 | - | 303.80 | 2.76 | Arboreal | Specialist/Forest | Critically Endangered |
|  | *Alouatta belzebul* | - | 0 | - | 2145.53 | 6.40 | Arboreal | Generalist/Forest | Vulnerable |
| Carnivora | *Leopardus pardalis* | 11.66 | 0 | 11.57 | 13.89 | 11.25 | Terrestrial | Generalist/Forest | Least Concern |
|  | *Leopardus wiedii* | 1.58 | - | 1.58 | - | 4.50 | Scansorial | Generalist/Forest | Near Threatened |
|  | *Puma concolor* | 0 | - | 5.09 | - | 29.00 | Terrestrial | Specialist/Forest | Least Concern |
|  | *Puma yagouaroundi* | 1.58 | - | 3.34 | - | 7.00 | Terrestrial | Generalist/Forest | Least Concern |
|  | *Panthera onca* | 0 | 72.91 | 8.15 | 26.70 | 55.00 | Terrestrial | Specialist/Forest | Near Threatened |
|  | *Cerdocyon thous* | 170.85 | 1365.45 | 0 | 0 | 7.40 | Terrestrial | Generalist/Open area | Least Concern |
|  | *Speothos venaticus* | - | 9.64 | - | 12.82 | 6.00 | Terrestrial | Specialist/Forest | Near Threatened |
|  | *Eira barbara* | 0 | 19.28 | 7.92 | 62.37 | 4.85 | Scansorial | Generalist/Forest | Least Concern |
|  | *Galictis vittata* | 7.89 | 28.92 | 0 | 0 | 2.50 | Terrestrial | Generalist/Forest | Least Concern |
|  | *Nasua nasua* | 3.22 | 156.25 | 98.79 | 865.67 | 4.50 | Scansorial | Generalist/Forest | Least Concern |
|  | *Potos flavus* | - | 0 | - | 13.89 | 2.20 | Arboreal | Generalist/Forest | Least Concern |
|  | *Procyon cancrivorus* | 21.80 | 72.91 | 0 | 12.82 | 6.25 | Scansorial | Generalist/Forest | Least Concern |
| Perissodactyla | *Tapirus terrestris* | 0 | 15.59 | 34.36 | 80.32 | 225.00 | Terrestrial | Specialist/Forest | Vulnerable |
| Artiodactyla | *Pecari tajacu* | 0 | 95.07 | 67.46 | 331.58 | 24.00 | Terrestrial | Generalist/Forest | Least Concern |
|  | *Tayassu pecari* | 0 | 0 | 51.63 | 290.98 | 34.00 | Terrestrial | Generalist/Forest | Vulnerable |
|  | *Mazama americana* | 0 | 0 | 37.09 | 68.37 | 27.50 | Terrestrial | Specialist/Forest | Data Deficient |
|  | *Mazama nemorivaga* | 3.50 | 0 | 85.71 | 41.86 | 15.00 | Terrestrial | Specialist/Forest | Least Concern |
| Rodentia | *Guerlinguetus aestuans* | 1.75 | 0 | 12.05 | 188.21 | 0.19 | Scansorial | Specialist/Forest | Least Concern |
|  | *Hydrochoerus hydrochaeris* | 1.75 | - | 1.59 | - | 50.00 | Semi aquatic | Generalist/Forest | Least Concern |
|  | *Cuniculus paca* | 1.58 | - | 43.18 | - | 9.35 | Terrestrial | Specialist/Forest | Least Concern |
|  | *Dasyprocta prymnolopha* | 0 | 230.65 | 389.06 | 1516.68 | 4.96 | Terrestrial | Specialist/Forest | Least Concern |

* Considering habitats preferable, not exclusive
