# Supplementary material for: Oil palm monoculture induces drastic erosion of an Amazonian forest mammal fauna
Source: PLoS One. 2017 Nov 8;12(11):e0187650. doi: 10.1371/journal.pone.0187650 (PMC5695600; doi:10.1371/journal.pone.0187650)
Supplement: S2 Table — The results include degrees of freedom (df); t-value (t); and p-value (p). (DOCX) [file pone.0187650.s008.docx]

**S2 Table.** Statistical results of Student´s paired t-tests to examine differences in total species Richness, total numerical Abundance, total Biomass, and Evenness values of the assemblages across habitat types on paired transects in either Oil Palm Plantation or Primary Forest. The results include degree of freedom (df); t-value (*t*); and *p*-value (*p*).

| Community patterns | LCT 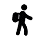 | | | CT 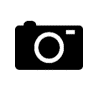 | | |
| --- | --- | --- | --- | --- | --- | --- |
|  | df | *T* | *p* | df | *t* | *p* |
| Richness | 7 | 5.39 | 0.001 | 7 | 3.74 | 0.007 |
| Abundance | 7 | 6.36 | 0.000 | 7 | 3.75 | 0.007 |
| Biomass | 7 | 4.26 | 0.003 | 7 | 4.21 | 0.003 |
| Evenness | 7 | -0.41 | 0.687 | 7 | 0.209 | 0.209 |
